# Nanorods with multidimensional optical information beyond the diffraction limit

**DOI:** 10.1038/s41467-020-19952-x

**Published:** 2020-11-27

**Authors:** Shihui Wen, Yongtao Liu, Fan Wang, Gungun Lin, Jiajia Zhou, Bingyang Shi, Yung Doug Suh, Dayong Jin

**Affiliations:** 1 Institute for Biomedical Materials & Devices (IBMD), Faculty of Science, University of Technology Sydney, Ultimo, NSW 2007 Australia; 2 Centre for Motor Neuron Disease Research, Department of Biomedical Sciences, Faculty of Medicine and Health Sciences, Macquarie University, Sydney, NSW 2109 Australia; 3 Laboratory for Advanced Molecular Probing, Research Center for Bio Platform Technology, Korea Research Institute of Chemical Technology, Daejeon, 34114 South Korea; 4 School of Chemical Engineering, SungKyunKwan University, Suwon, 16419 South Korea; 5 UTS-SUStech Joint Research Centre for Biomedical Materials & Devices, Southern University of Science and Technology, Shenzhen, Guangdong, 518055 PR China

**Keywords:** Chemical engineering, Materials for optics, Synthesis and processing, Nanoparticles

## Abstract

Precise design and fabrication of heterogeneous nanostructures will enable nanoscale devices to integrate multiple desirable functionalities. But due to the diffraction limit (~200 nm), the optical uniformity and diversity within the heterogeneous functional nanostructures are hardly controlled and characterized. Here, we report a set of heterogeneous nanorods; each optically active section has its unique nonlinear response to donut-shaped illumination, so that one can discern each section with super-resolution. To achieve this, we first realize an approach of highly controlled epitaxial growth and produce a range of heterogeneous structures. Each section along the nanorod structure displays tunable upconversion emissions, in four optical dimensions, including color, lifetime, excitation wavelength, and power dependency. Moreover, we demonstrate a 210 nm single nanorod as an extremely small polychromatic light source for the on-demand generation of RGB photonic emissions. This work benchmarks our ability toward the full control of sub-diffraction-limit optical diversities of single heterogeneous nanoparticles.

## Abstract (web summary)

Development of functional nanostructures can enable a range of applications in imaging and nanoscale science. Here, the authors fabricate and characterize complex heterogeneous nanorods with diverse, tunable sub-wavelength structures.

## Introduction

The frontier in nanomaterials engineering is to realize their composition control with atomic-scale precision, so as to fabricate nanoscale structures with desirable morphological, interfacial, and physical properties^1,2^. The control in uniformity and tuning the diversities of single luminescent nanoparticles, dot to dot, has already enabled many quantitative measurements and high-performance nanophotonic devices^3,4^. The next level of challenges and opportunities should be the controlled growth, manipulation, and characterization of the hybrid and heterogeneous nanostructures that can incorporate multiple functionalities. But the nanoscale optical properties of such nanostructures are hardly accessible, due to the Abbe diffraction limit that confines the best axial and lateral resolutions of far-field optical microscopy worse than 200 nm and 500 nm, respectively^5^.

Due to a wealth of electronic transitions within the 4f electron shells of lanthanide ions, nanostructures doped with lanthanide ions can form a unique class of functional optical devices^6,7^. Among them, spherical upconversion nanoparticles (UCNPs) have been created with many unique optical properties, including tuneable colors^8^, multiplexed lifetimes^9,10^, long-distance energy migration^11^, amplified stimulated emissions^12–16^ and their responses to external fields of temperature^17^ and mechanical force^18^, which enables many novel applications, including full-color displays^8^, solar energy harvesting^19^, security inks^9^, biomolecular sensing^20^, force sensing^21^, nanothermometry^22,23^, fluorescence microscopy^12^, optical multiplexing^9,24^, deep-tissue optogenetics^25^, multimodal bio-imaging^26,27^, and light-triggered drug delivery^28,29^.

Here, we realize the artful control in the single-axis epitaxial growth of uniform lanthanide-doped heterogeneous nanorods with high-dimensional optical signatures. Each section along the nanorod structure can be controlled to display tunable emissions and resolved by the super-resolution microscopy in four optical dimensions, including emission wavelength, lifetime, excitation wavelength, and excitation power dependency. Moreover, we demonstrate a 210 nm single nanorod as the smallest polychromatic light source for the on-demand generation of RGB photonic emissions.

## Results

### Single-axis growth of heterogeneous nanorods

**Fig. 1:**
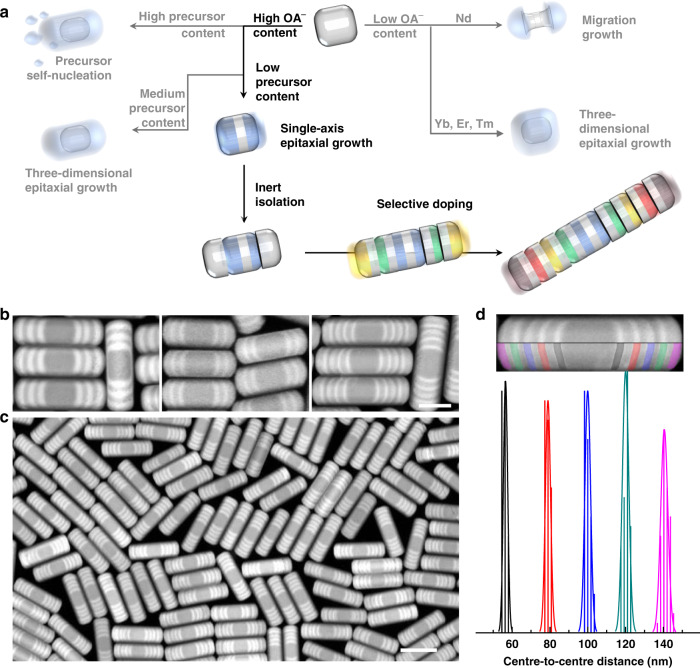
Single-axis epitaxial controlled growth of heterogeneous nanorods. **a** Schematic diagram to illustrate the route that leads to the formation of the nanorods. Conditions, including the well-controlled amount of surfactant concentration (OA^−^) and the relatively low concentration of shell precursor, have to be met, otherwise, nanoplates, nanodumbbells, and small nanoparticles from precursor self-nucleation, will form. **b** High-angle annular dark-field scanning transmission electron microscopy (HAADF-STEM) images of three typical heterogeneous nanorods, including 10-segment NaYF_4_-NaErF_4_(10 nm)-NaYF_4_(5 nm)-NaErF_4_(10 nm)-NaYF_4_(5 nm)-NaErF_4_(10 nm), 10-segment NaYF_4_-NaErF_4_(5 nm)-NaYF_4_(5 nm)-NaErF_4_(5 nm)-NaYF_4_(5 nm)-NaErF_4_(5 nm), and 18-segment NaYF_4_-NaErF_4_(5 nm)-NaYF_4_(5 nm)-NaErF_4_(5 nm)-NaYF_4_(5 nm)-NaErF_4_(5 nm)-NaYF_4_(5 nm)-NaErF_4_(5 nm)-NaYF_4_(5 nm)-NaErF_4_(5 nm) nanorods, scale bar is 50 nm. **c** Large area HAADF-STEM image of 18-segment heterogeneous nanorods, scale bar is 100 nm. **d** Distance statistics of five pairwise NaErF_4_ segments (center-to-center of each pairwise with different color markers) of the heterogeneous nanorods in **c**.

Figure 1a summarizes the key procedure and critical conditions that lead to the heterogeneous nanorods. The principle that directs the controlled epitaxial growth is based on the fact that the surfactants—oleic acid molecules (OAH) prefer to be attached to the (001) facet while the oleic acid anions (OA^−^) bind more firmly onto the (100)/(010) facets of a β-NaYF_4_ nanocrystal^30^. Here we find that using a high OA^−^/OAH ratio (1:2) and at the slightly increased reaction temperature (310 °C), the crystal growth rate on the (001) facet can be a lot faster than that on the (100)/(010) facets, so that heterogeneous nanorods can be formed. For single-axis growth of nanorods, the amount of the precursor should be kept sufficiently low. For instance, when the concentration of the NaYF_4_ shell precursor is kept at a relatively high level (0.732 μmol/mL), the growth rates of 1.68 atomic layers per min on the (001) facet versus 0.26 atomic layer per min on the (100)/(010) facets were observed (Supplementary Note 2). If the supply of precursors is less than the demand in the epitaxial growth from the two (001) facets of the core nanocrystals (0.235 μmol/mL), desirable single-axis growth could be achieved (Supplementary Figs. 2–4). Notably, in the heterogeneous epitaxial growth process of nanorods, high OA^−^ concentration is critical to avoid the formation of the dumbbell and core@shell structures of NaNdF_4_ and NaLnF_4_ (Ln = Yb, Er, Tm), respectively (Supplementary Figs 5 and 6).

### Morphology uniformity of heterogeneous nanorods

By achieving such strict control, the HAADF-STEM micrographs (Fig. 1b) show the fabrication of a series of heterogeneous nanorod structures with length tunable segments (e.g., 5 nm and 10 nm) by adjusting the time for precursor injection. Also, the number of segments can be arbitrarily controlled by using the different composition of precursors. Figure 1c shows a large area HAADF-STEM image of a highly uniform 18-segment nanorod structure, having each segment with a fixed length of 5 nm. The invariant width of 42 nm in diameter for the multi-segment heterogeneous nanorods further confirms the absolute control in the single-axis growth (Supplementary Fig. 7). Remarkably, the statistical analysis shown in Fig. 1d further confirms that these nanorods are exceptionally uniform even in the length of each segment. By calculating the precursor injection speed and the thickness of each segment, it is shown that the growth rates of both NaYF_4_ and NaErF_4_ on the (001) facet are one atomic layer per minute in current reaction system. The use of the optically inert segments of NaYF_4_ in the heterogeneous nanorods can minimize the diffusion of optically active ions, and therefore allow arbitrary integration of multiple orthogonal optical responses across the nanorods.

### Super-resolution imaging of the heterogeneous optical segments within a single nanorod

**Fig. 2:**
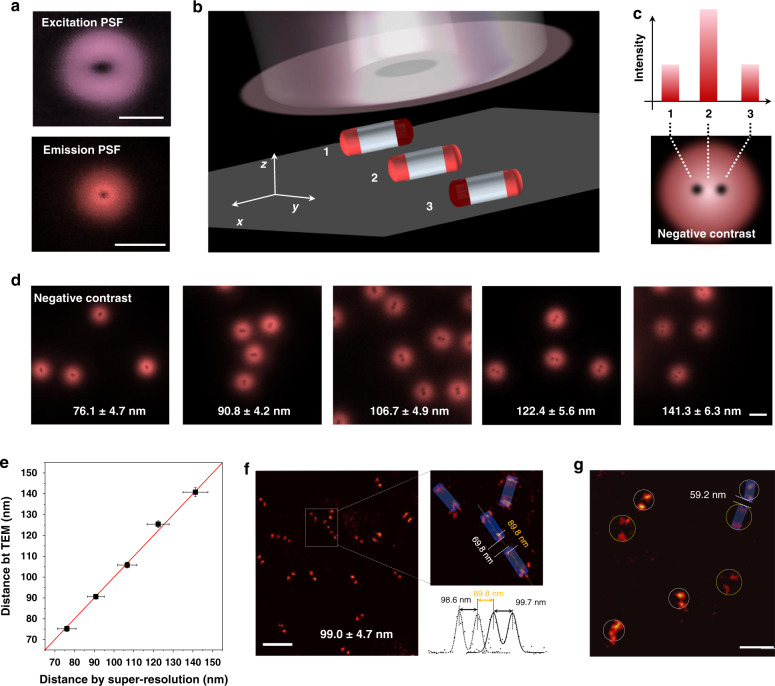
Super-resolution imaging of nanorods with different length. **a** The pattern of the donut-shaped illumination of excitation point spread function (PSF) and the pattern of the resulted emission PSF after scanning across a single UCNPs. **b** Illustration of a process using a donut-shaped excitation beam to scan and resolve the heterogeneous structure of a single upconversion nanorod. **c** The corresponding luminescence intensities at the three typical positions to resolve the final negative-contrast super-resolution image of a nanorod. **d** The raw data of negative-contrast super-resolution images of five types of heterogeneous upconversion nanorods with tuneable lengths between the optically active components. **e** The average center-to-center distance and standard deviation of two actively doped segments of five types of heterogeneous upconversion nanorods measured from TEM and super-resolution imaging, data are shown as the mean ± SD. **f** Super-resolution image of the high concentration nanorods with an active segmental distance of 99.0 nm. **g** Super-resolution image of the mixture of two types of nanorods with active segmental distances of 90 nm and 122 nm, respectively. Pixel sizes in **d**, **f**, and **g** are 10 nm. Scale bars in **a**, **d**, **f**, and **g** are 500, 500, 400, and 250 nm, respectively.

To resolve the optical information of different segments within a single nanorod, we employ an annular excitation profile (donut-shape) of the illumination beam in a typical confocal microscopy setup. As illustrated in Fig. 2a, the size of the donut beam is relatively large with a full-width at half-maximum (FWHM) around 300 nm, set by the diffraction limit of the excitation point spread function (PSF). By taking advantage of both the non-linear optical responses of upconversion luminescence and its low saturation intensity levels, the emission PSF can produce a much smaller dark area (negative contrast), with the FWHM down to around 29 nm (Supplementary Fig. 8b, c), which allows the emission saturation mode to be used for super-resolution imaging^31^. Note that instead of the upconversion stimulated emission depletion (STED) super-resolution microscopy configuration^12–14,32^ that only works well for depleting the emissions from UCNPs highly doped with Tm^3+^ ions, our current method is a lot simpler and broadly compatible with different lanthanide emitters, doping concentrations, and emission bands^31^. The design of a single beam donut illumination avoids the sophisticated system alignment and temporal synchronization of both Gaussian excitation and donut depletion beams required by STED.

We first image a set of sub-diffraction-limit nanorods with a pair of identical active segments grown on both ends. As shown in Fig. 2b, by a donut-shaped excitation beam scan across the heterogeneous nanorods, a negative contrast will be generated when any one of the optically active units is positioned within the donut beam, so that the final image of a single nanorod will be presented as two negative-contrast spots (1 and 3), as shown in Fig. 2c. As based on the mathematical calculation of Abbe diffraction limit the spatial resolution of our emission saturation super-resolution microscopy is 29 nm (Supplementary Fig. 8b, c), the limit in resolving the two active segments needs to be longer than the distance of 50 nm (Supplementary Fig. 8d–f). As the resolution is also dependent on the signal-to-noise ratio of the images, we set the length of each functional segment as 20 nm to achieve sufficiently bright with a signal-to-noise ratio larger than 5. To experimentally verify the optical resolving ability, we further fabricate five batches of distance-tuneable nanorods (Supplementary Fig. 9). As the centroids of the dark spots in each negative-contrast super-resolution image (Fig. 2d and Supplementary Fig. 11) can be used to localize the active segments in the nanorods, the super-resolution images of the five types of representative nanorods show the central distance increasing from 76 to 141 nm, which is highly consistent with the TEM results (Fig. 2e). Taking account of the length of the 20 nm active segments, the separation distance between active units (the length of the optically inert NaYF_4_ section) is 55 to 120 nm (Supplementary Fig. 9). These results confirm the spatial resolving power of our single-beam super-resolution approach as around 55 nm, which is comparable to the reported super-resolution techniques used for nanomaterials^33–36^. Notably, due to the nonlinear power dependence property of the emission PSF, choosing an appropriate excitation power is critical in optimizing the localization accuracy (Supplementary Fig. 12).

Moreover, as shown in Fig. 2f and Supplementary Fig. 13a, the single-donut-beam approach can be used to resolve the clusters of multiple nanorods. Though the focus of our work is to resolve the sub-segments of optical diversities within a single nanorod and selectively excite these sub-units, the heterogeneously coded nanorods may become a new kind of fluorescent tags, and the orientation information inside the bi-punctate nanorods structure can be used for super-resolution imaging and single nanoparticle localization (tracking). Ideally, the distance of any two nearby nanorods should be greater than the bi-punctate spot separation to determine which pair of spots belong to which nanorod. This makes our nanorods suitable for low-density labeling applications. The orientation information inside the bi-punctate nanorods structure presents an opportunity for future applications where the dipole orientation information in three coordinates are desired^37^. The other challenge for these nanorods to be used as the fluorescent tag is that the size (typically > 70 nm) of these rods are relatively too large for intracellular applications^5^. By taking advantage of the pair structure of identical segments on each single nanorod, the sample distance of as close as 69.8 nm can be resolved. Furthermore, as shown in Fig. 2g and Supplementary Fig. 13b, the super-resolution approach can successfully resolve two populations of nanorods with active segmental distances of 90 nm and 122 nm, respectively. The distance between two types of nanorods as close as 59.2 nm has been resolved with the low sample density. The signal-to-noise ratio can be further improved by increasing the volume and brightness of each segment of the heterogeneous nanorods^38^.

### Selective activation of nanorods’ segments

**Fig. 3:**
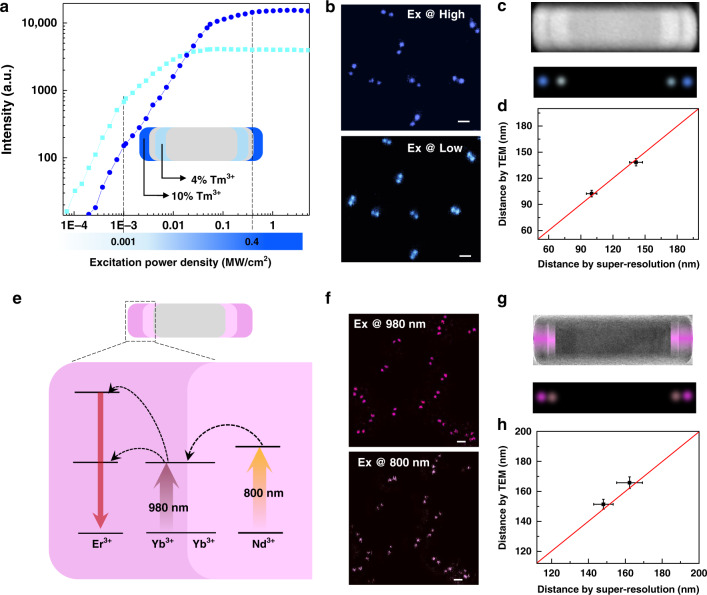
Super-resolution decoding nanorods with different optical dimensions. **a** The scheme using the excitation-power-dependent property to activate the different segments of a purpose-designed nanorod. The upconversion emissions, generated from the segment doped with relatively higher concentration (10% Tm^3+^), can be turned on at higher excitation power density, compared with that from lower doped (4% Tm^3+^) segments. **b** Super-resolution images of the functional segments selectively decoded by high and low power density conditions. **c** HAADF-STEM image and corresponding two-channel super-resolution image. **d** Statistical analysis of the distance distributions of different sections measured by TEM images and super-resolution images, data are shown as the mean ± SD. **e** The scheme using the long-distance energy migration property to activate the nearby segments for decoding the location of different sensitizers. **f** Super-resolution images of the functional segments selectively decoded by 980 nm and 808 nm excitation. **g** TEM image and corresponding two excitation-wavelength-channel super-resolution images. **h** Statistical analysis of the distance distributions of different sections measured by TEM images and super-resolution images, data are shown as the mean ± SD. Pixel sizes in **b** and **f** are 10 nm. Scale bars are 200 nm. High-resolution images of **b** and **f** are shown in Supplementary Fig. 18.

The optically active units doped by lanthanide ions, typically with characteristic ‘ladder-like’ multiple excited states, can generate near-infrared (NIR), visible, and ultraviolet luminescence with the sharp spectrum, large (anti-)Stokes shift, and inherent long lifetime^11,39–41^. As the emission saturation super-resolution microscopy used in this work is not limited by the types of lanthanide emitters, we can code and decode the optically functional segments of nanorods by using a diverse range of choices of lanthanide co-dopants. First, it is a rather straight-forward strategy by collecting the emission information from different wavelength windows, i.e., NIR and red emissions from Tm^3+^ and Er^3+^ emitters (Supplementary Fig. 14), respectively. Then, we also evaluate the capability of using different concentrations of emitters in each segment. The power-dependent property^39,42^ (Fig. 3a) allows the selectively activating of the segments of nanorods beyond the diffraction limit. Selective activation of two sections doped by the relatively lower concentration of emitters (4% Tm^3+^) and a higher concentration of emitters (10% Tm^3+^) can be achieved by using low and high power densities of 980 nm excitation, respectively (Fig. 3b). The distances of different active pairwise segments are consistent with that from the TEM characterization (Fig. 3c, d and Supplementary Fig. 15). Notably, the emission difference from the two segments can be further resolved by the time-resolved mode, as the highly doped sections display a much shorter lifetime (Supplementary Fig. 16).

### Visualizing interfacial energy transfer within a single nanorod

We further resolve the information encoded through interfacial energy transfer and migration across the heterogeneous upconversion nanorod. Here, by designing the Yb^3+^/Er^3+^ co-doped upconversion segment closely packed next to the Yb^3+^/Nd^3+^ co-doped sensitization segments (Fig. 3e), we show the selective activation and decoding of these neighboring segments. This has been achieved by either exciting the upconversion segment using the 980 nm donut illumination or the nearby sensitization segments by using the 808 nm donut illumination, where the energy migration from Nd^3+^ → Yb^3+^ → Yb^3+^ → Er^3+^ occurs across the interface. Because the 808 nm donut excitation only examines where the material absorbs, we can localize the photon-sensitization segments with Nd^3+^ as dopants (Supplementary Fig. 17). Following the same principle, the 980 nm donut excitation is responsible for diagnosing the location of Yb^3+^ ions. The super-resolution images (Fig. 3f) clearly reveal both the Nd^3+^ and Yb^3+^ sensitization segments with the center-to-center distance around 7.2 nm, which is further verified by the TEM characterization (Fig. 3g, h and Supplementary Fig. 17). Therefore, we precisely localize the position of closely packed sensitizer sections and visualize the energy transfer effect at the nanoscale. The developed method has the potential to study the interfacial energy transfer across two neighboring segments, which opens the door for fine tunings of the emission^11^ and excitation^43^ wavelengths, emission intensity^44^, and lifetimes^9^.

### Sub-diffraction-limit RGB-switchable pixel

**Fig. 4:**
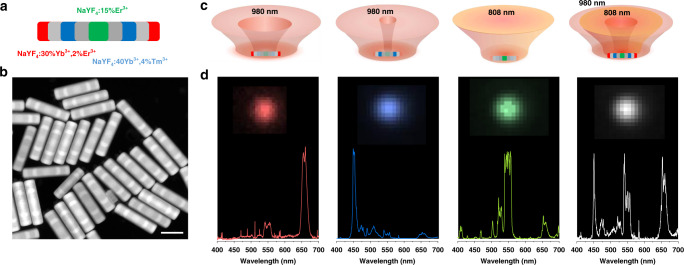
On-demand generation of RGB colors from a single nanorod. **a** The scheme to integrate multiple segments with different dopants, each responsible for emitting red, blue, and green colors. **b** HAADF-STEM image of the heterogeneous 210 nm optical rods, scale bar is 100 nm. **c** Diagrams of the bright upconversion emissions with switchable colors in response to the illumination patterns of a Gauss–Laguerre mode donut profile (980 nm) and a Gaussian excitation profile (808 nm). **d** Wide-field microscopy (pseudocolor) images of a single rod and their corresponding luminescence spectra under the different illumination conditions in **c**.

The ability of selective activations of high-dimensional emission diversities from a single nanorod can further create a sub-diffraction-limit light-emitting pixel, where RGB emissions can be arbitrarily switchable, in response to the specific excitation wavelength and illumination pattern, as illustrated in Fig. 4. This experiment takes advantage that the tunable donut and Gaussian excitation profiles at 980 nm and 808 nm can be commensurate to the bilateral symmetry of the rod stratification. When a relatively large diameter of the 980 nm donut excitation is used, the pair of Red units (NaYF_4_:30%Yb^3+^, 2%Er^3+^) on the end of the rod can be selectively illuminated. The smaller size of 980 nm donut excitation activates the pair of Blue units (NaYF_4_:40%Yb^3+^, 4%Tm^3+^) in the middle of the nanorods. In addition, the 808 nm confocal spot of excitation is responsible for illumining the central Green emission unit (NaYF_4_:15%Er^3+^), since neither of the Red unit or the Blue unit has the effective absorbance at 808 nm. White emission can be generated through the combined use of the 808 nm confocal and 980 donut excitations. These results show our ability to selectively activate different optical units in the heterogeneous nanostructures, which opens the door to build more sophisticated nanophotonic devices that can on-demand display a set of high-dimensional digitized photonic emissions.

## Discussion

We presented here the advances in the controlled synthesis of heterogeneous nanorods, methods, and instrumentation for super-resolution characterizations of optically active segments within a single nanorod. We demonstrated their potentials in the sub-diffraction-limit optical characterization and selective activation of doping units with a set of high-dimensional differentiable photonic emissions. Both the artful control of heterogeneous nanostructures with exceptionally high uniformity and the precise discerning of diverse non-linear responses at super-resolution are the key technology enablers. While this work provides new scopes for controlled fabrication and sub-diffraction-limit characterizations of the anisotropic heterogeneous nanomaterials, we anticipate many photophysics to be discovered from the new library of geometrically distinct nanostructures, including the dynamics and directions of energy transfer process within the heterogeneous nanomaterials^45^.

## Methods

### Materials

YCl_3_·6H_2_O (99.99%), YbCl_3_·6H_2_O (99.9%), ErCl_3_·6H_2_O (99.995%), TmCl_3_·6H_2_O (99.99%), NH_4_F (>98%), NaOH (>99.9%), KOH (99.9%), Oleic acid (OA, 90%), and 1-octadecene (ODE, 90%) were purchased from Sigma-Aldrich and used as received without further purification.

### Synthesis of NaYF_4_ core nanoparticles

NaYF_4_ nanoparticles were synthesized through the coprecipitation method. In a typical experiment, 2 mmol YCl_3_·6H_2_O were added to a 100 mL flask containing oleic acid (OA, 12 mL) and 1-octadecene (ODE, 30 mL). The mixture was heated to 170 °C under argon for 30 min to obtain a clear solution and then cooled down to about 50 °C, followed by the addition of 10 mL methanol solution of NH_4_F (8 mmol) and NaOH (5 mmol). After stirring for 30 min, the solution was heated to 80 °C under argon for 20 min to remove methanol, and then the solution was further heated to 310 °C for another 90 min. Finally, the reaction solution was cooled down to room temperature, and nanoparticles were precipitated by ethanol and washed with cyclohexane, ethanol, and methanol 3 times to get the NaYF_4_ core nanoparticles.

### Synthesis of NaYF_4_ nanorods

NaYF_4_ nanorods were synthesized by longitudinal growth of NaYF_4_ shell precursors onto the NaYF_4_ core nanoparticles via a successive layer-by-layer hot-injection protocol. Firstly, shell precursors were prepared. In total 1.5 mmol YCl_3_·6H_2_O were added to a 50 mL flask containing 6 mL OA and 15 mL ODE. The mixture was heated to 170 °C under argon for 30 min to obtain a clear solution and then cooled down to about 50 °C, followed by the addition of 12 mL methanol solution of NH_4_F (6.0 mmol), NaOH (3.75 mmol), and KOH (3 mmol). After stirring for 30 min, the solution was heated to 80 °C under argon for 30 min to remove methanol, and then the solution was further heated to 150 °C for another 20 min. Finally, the reaction solution was cooled down to room temperature and labeled as NaYF_4_ shell precursors.

For the longitudinal growth, 0.2 mmol NaYF_4_ core particles were added to a 50 mL flask containing 3.6 mL OA, 9.2 mL ODE, and 40 mg NaOH. The mixture was heated to 170 °C under argon for 30 min, and then the solution was further heated to 310 °C. After that, 0.04 mL of NaYF_4_ shell precursors were injected into the reaction mixture and ripened at 310 °C for 1 min followed by the same injection and ripening cycles for different times to get the nanorods with different length. Finally, the reaction solution was cooled down to room temperature and the formed nanorods were purified according to the procedures used for the purification of NaYF_4_ core particles.

### Morphology characterization

The morphology of the formed materials was characterized via transmission electron microscopy (TEM) imaging (Philips CM10 TEM with Olympus Sis Megaview G2 Digital Camera) with an operating voltage of 100 kV. The samples were prepared by placing a drop of a dilute suspension of nanocrystals onto copper grids. High-angle annular dark-field (HAADF) scanning transmission electron microscope (STEM) images were collected with a Talos F200X S/TEM (Thermo Scientific™).

## Supplementary information

Peer Review File

Supplementary Information

## Data Availability

All the relevant data supporting the findings of this study are available with the paper and its supplementary information files. Source data are provided with this paper.

## Source data

Source Data
