# Supplementary material for: Nanorods with multidimensional optical information beyond the diffraction limit
Source: Nat Commun. 2020 Nov 27;11:6047. doi: 10.1038/s41467-020-19952-x (PMC7695702; doi:10.1038/s41467-020-19952-x)
Supplement: Supplementary file 2 — Supplementary Information [file 41467_2020_19952_MOESM2_ESM.pdf]

Supplementary Information for

Nanorods with multidimensional optical information beyond the diffraction limit

Shihui Wen[1], Yongtao Liu[1], Fan Wang[1], Gungun Lin[1], Jiajia Zhou[1], Bingyang Shi[2], Yung Doug Suh[3,4], Dayong Jin[1,5]*

[1] Institute for Biomedical Materials & Devices (IBMD), Faculty of Science, University of Technology Sydney, Ultimo, NSW 2007, Australia

[2] Centre for Motor Neuron Disease Research, Department of Biomedical Sciences Faculty of Medicine and Health Sciences, Macquarie University, Sydney, NSW, 2109 Australia

[3] Laboratory for Advanced Molecular Probing, Research Center for Bio Platform Technology, Korea Research Institute of Chemical Technology, Daejeon, South Korea

[4] School of Chemical Engineering, SungKyunKwan University, Suwon 16419, South Korea

[5] UTS-SUStech Joint Research Centre for Biomedical Materials & Devices, Southern University of Science and Technology, Shenzhen, Guangdong 518055, P. R. China

These authors contributed equally to this work: Shihui Wen, Yongtao Liu.

*Correspondence to: D. Jin (dayong.jin@uts.edu.au).

Contents

Supplementary Note 1. Super-resolution characterization. 3

Supplementary Note 2. Calculating the growth rates on different facets of the nanocrystals. 5

Supplementary Note 3. One-dimensional growth of $NaYF_4$ and $NaErF_4$ onto the $NaYF_4$ core nanoparticles. 10

Supplementary Note 4. Design and synthesis of optical nanorods with different length........... 13

Supplementary Note 5. Design and synthesis of colour-based nanorods.................................. 19

Supplementary Note 6. Design and synthesis of excitation-power-based nanorods................. 21

Supplementary Note 7. Design and synthesis of excitation-wavelength-based nanorods.......... 24

Supplementary Note 8. Design and synthesis of RGB colors nanorods. 27

Supplementary References... 28

Supplementary Note 1. Super-resolution characterization.

The optical characterization was performed on a commercial microscope (Olympus IX73) equipped with a 3-axis closed loop piezo stage (Nano Nano-LP Series, Mad city labs) and free space design of optics. A schematic drawing of the experimental setup was presented in **Supplementary Figure 1**. A single-mode fiber-coupled 980 nm diode laser (BL976-PAG900, controller CLD1015, Thorlabs; maximum output power 900 mW) was used as the excitation source. After collimation, the excitation beam was transmitted through the first half wave plate (HWP, WPH05M-980, Thorlabs) and polarized beam splitter (PBS, CCM1-PBS252/M, Thorlabs). This allowed precisely adjusting the excitation power by rotating HWP electronically. The vortex phase plate was used to generate a doughnut-shaped point spread function (PSF) at the focal plane. A 4f optical system (L3 and L5) was used to transfer the image plane into the back aperture of the objective lens. The purpose of the second HWP was to control the polarization of excitation beam. After further improving the polarization ratio by a glan Taylor (DGL10, Double Glan-Taylor Polarizer, Thorlabs), the excitation beam was transmitted through a long-pass dichroic mirror (ZT915lpxr, Chroma), then reflected by a second short-pass dichroic mirror (T750spxrxt, Chroma), and focused through a high numerical aperture objective (UPlanSApo, 100×/1.40 oil, Olympus) to the sample slide. The long-pass dichroic mirror was used to combine the 980 nm beam with the 808 nm excitation beam from a polarization-maintaining fiber-coupled diode laser (LU0808M250, 808nm Single Mode Laser Module, Lumics). Another 808 nm excitation laser shared similar beam path as that for the 980 nm laser, in relation to the control of its power and polarization. The quarter-wave plate (QWP, WPQ05M-980/808, Thorlabs) was adopted to transform the excitation beam from linear polarization to circular polarization to obtain optical super-resolution images. Photoluminescence signal from the sample was collected by the same objective, then directed to the detection unit by the second dichroic mirror. Before being coupled into a multi-mode fiber (MMF, M24L02, Thorlabs) for detection, the emission signals went through either a bandpass (BPF, ET805/20M, ET670/50M, ET460/50M, Chroma) or a short pass filter (SPF, FF01-842/SP-25/ FF01-750/SP-25, Semrock) to completely remove the excitation light. A single-photon counting avalanche photodiode (APD, SPCM-AQRH-14-FC, Excelitas) was used for (time-gated) detection, controlled by a Labview program. The MMF could also be connected to a spectrometer (Shamrock 193i Spectrograph, Andor) equipped with an EMCCD

detector (iXon Ultra, Andor). Each scanning step was set as 10 nm and pixel dwell times were adjusted to be 3 ms.

To prepare a sample slide, a cover slip was washed with pure ethanol and then Milli-Q water under ultrasonication. 10 μL of the upconversion nanorods (diluted to 0.01 mg/mL in cyclohexane) were dropped onto the surface, which was immediately washed with 500μL cyclohexane twice for optimizing the particle concentration on the slip. After being air-dried, the cover slip was put over a clean glass slide.

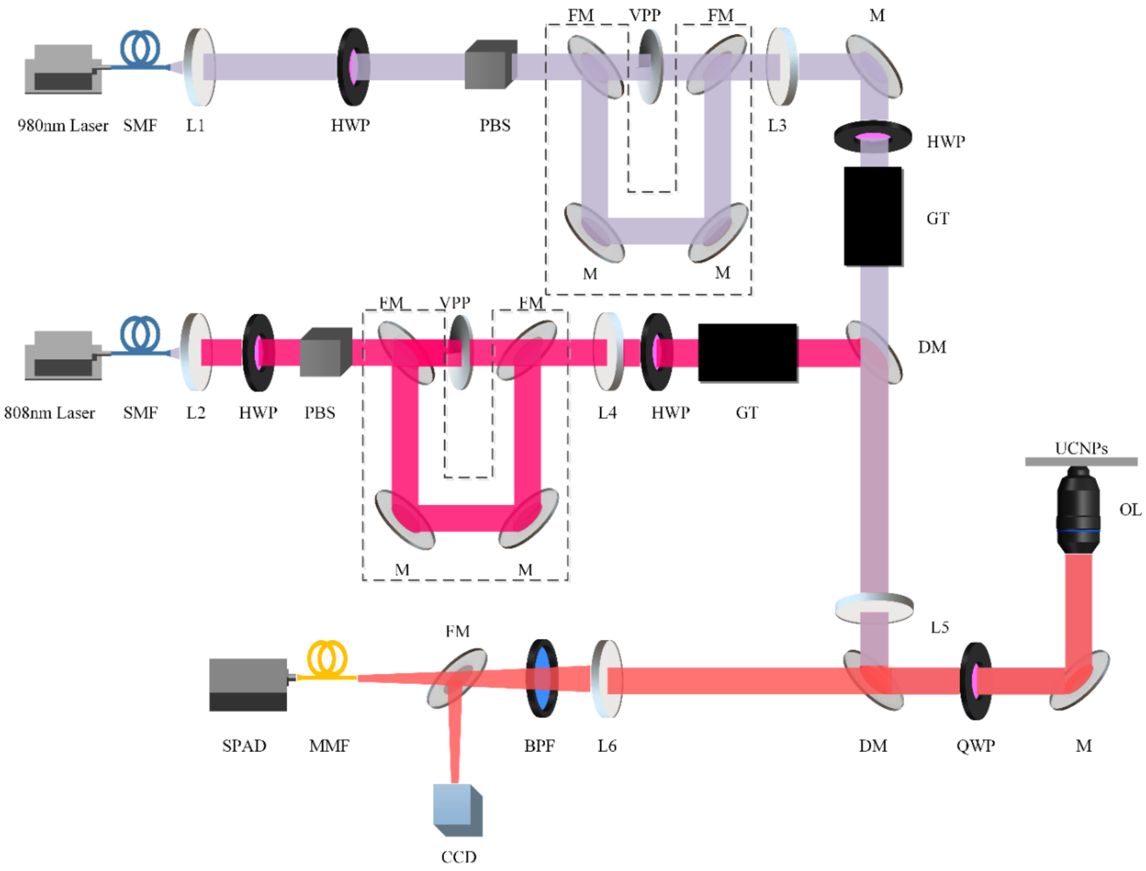

Supplementary Figure 1. Scheme of the purpose-built optical measurement setup. OL: Objective lens; M: mirror; FM: flexible mirror; QWP: quarter-wave plate; HWP: half-wave plate; SMF: single-mode fiber; MMF: multi-mode fiber; PBS: polarized beam splitter; VPP: vortex phase plate; DM: dichroic mirror; BPF: band pass filter; SPAD: single-photon avalanche diode; CCD: charge coupled device. L1, L2, L6: collimation and collection lens; L3, L4, L5: 4f optical system lens; The dotted portion is designed for auxiliary confocal with two flexible mirrors to bypass the VPP in the main optical path.

Supplementary Note 2. Calculating the growth rates on different facets of the nanocrystals.

According to our recent report [1], surfactant ligands of oleic acid molecules (OAH) and oleic cations (OA^-) preferably bond onto the (001) and (100)/(010) facets of a β-$NaYF_4$ nanocrystal, respectively. The dynamic roles of OAH and OA^- surfactant molecules depend strongly on their synthesis environments and binding strength to the rare earth ions (precursor). The surfactant of OAH has a relatively weak binding energy onto the (001) facet. When the environment becomes unstable, e.g. at increased temperature, OAH will be detached from rare earth ions so that new occupancies will be created for new atoms to be grown into. The surfactant of OA^- cations, however, have much stronger binding strength to the rare earth ions and strongly anchor onto the (100)/(010) facets, and effectively terminate the continuous growth even at high temperature. Therefore, under these specific conditions, one-dimensional epitaxial growth should be well under control.

To make one-dimensional epitaxial growth under control, the crystals growth kinetics on different facets were first studied. We employed the layer-by-layer hot injection method, which could make the concentration of the shell precursor relatively consistent during the growth process, to systematically optimize the effect of the concentration of the shell precursors on the facet-selective growth behaviour.

Epitaxial growth with 1.465 μmol/mL $NaYF_4$ precursors (high concentration). For the longitudinal growth of $NaYF_4$ with high concentration of the precursors, 0.2 mmol $NaYF_4$ core particles were added to a 50 mL flask containing 3.6 mL OA, 9.2 mL ODE, and 40 mg NaOH. The mixture was heated to 170 °C under argon for 30 min, and then the solution was further heated to 310 °C . After that, 0.25 mL of $NaYF_4$ shell precursors were injected into the reaction mixture and ripened at 310 °C for 1 min followed by the same injection and ripening cycles for 16 times to get the nanorods. Finally, the reaction solution was cooled down to room temperature and the formed nanorods were purified according to the procedures used for the purification of $NaYF_4$ core particles. As shown in **Supplementary Figure 2b**, apart from the formed nanorods, there are small nanoparticles formed by precursor self-nucleation. This indicates that the concentration of the precursor is too high to have the controlled epitaxial growth. Therefore, we reduced the precursor concentration to get uniform nanocrystals.

Epitaxial growth with 0.732 μmol/mL $NaYF_4$ precursors (medium concentration). For the longitudinal growth of $NaYF_4$ with medium concentration of the precursors, 0.2 mmol $NaYF_4$ core particles were added to a 50 mL flask containing 3.6 mL OA, 9.2 mL ODE, and 40 mg NaOH. The mixture was heated to 170 °C under argon for 30 min, and then the solution was further heated to 310 °C. After that, 0.125 mL of $NaYF_4$ shell precursors were injected into the reaction mixture and ripened at 310 °C for 1 min followed by the same injection and ripening cycles for 32 times. Finally, the formed nanorods were purified for TEM characterization. As shown in **Supplementary Figure 3**, there were uniform nanorods formed with the medium concentration of the precursors. The length of the nanocrystals increased from 37 nm to 56 nm with injection cycles of 32 times. But, the width of the nanocrystals also increased from 37 nm to 42 nm at the same time. This result indicates that part of the shell precursor could not bind to the preferred (001) facet but grow on the non-preferred one (100/010 facets). Through the calculation, there was 5 nm growth in the transversal direction while there was 19 nm increase in the longitudinal direction. For β-$NaYbF_4$ crystal, the hexagonal cylinder consists of the (001) facets at the ends and identical (100) and (010) facets around the cylinder sides. The Y^{3+} atoms form equilateral triangles with a length of 5.96 Å in the relaxed (001) surface (transversal direction), while rectangles are observed in the (100) and (010) surface with a shorter length of 3.53 Å (longitudinal direction). Therefore, the growth rate along the longitudinal direction is 1.68 atomic layers per min, while that for the transversal direction it is around 0.26 atomic layer per min under the medium precursor concentration.

To get the precise one-dimensional growth, it is better to reduce the amount of the available precursors equal or less than the needed for the (001) facets of the seed nanocrystals. 0.2 mmol $NaYF_4$ core particles is equal to 2.98×10^{14} 37-nm nanocrystals with the requirement of 0.003 mmol $NaYF_4$ shell precursor for one-layer growth at the (001) facets. As the volume of the reaction solution is around 12.8 mL, the concentration of the shell precursor should be around 0.235 μmol/mL.

Epitaxial growth with 0.235 μmol/mL $NaYF_4$ precursors (low concentration). For the longitudinal growth of $NaYF_4$ with low concentration of precursors, 0.2 mmol $NaYF_4$ core particles were added to a 50 mL flask containing 3.6 mL OA, 9.2 mL ODE, and 40 mg NaOH. The mixture was heated to 170 °C under argon for 30 min, and then the solution was further heated to 310 °C. After that, 0.04 mL of $NaYF_4$ shell precursors were injected into the reaction mixture

and ripened at 310 °C for 1 min followed by the same injection and ripening cycles for 100 times. Finally, the formed nanorods were purified for TEM characterization. As shown in **Supplementary Figure 4**, there were uniform nanorods formed with the low concentration of the precursors. Also, the length of the nanocrystals increased from 37 nm to 72 nm with injection cycles of 100 times, while the width of the nanocrystals is still around 37 nm. Through the calculation, there was 35 nm increase in longitudinal direction with the growth rate around one atomic layer per min. This indicates that the lower shell precursor in the reaction solution makes the one-dimensional growth well in control with the assistant of optimized OA- concentration and reaction temperature. In this case, both the width (depend on the size of the seeds) and length (depend on the injection cycles) of the nanorods could be well in control.

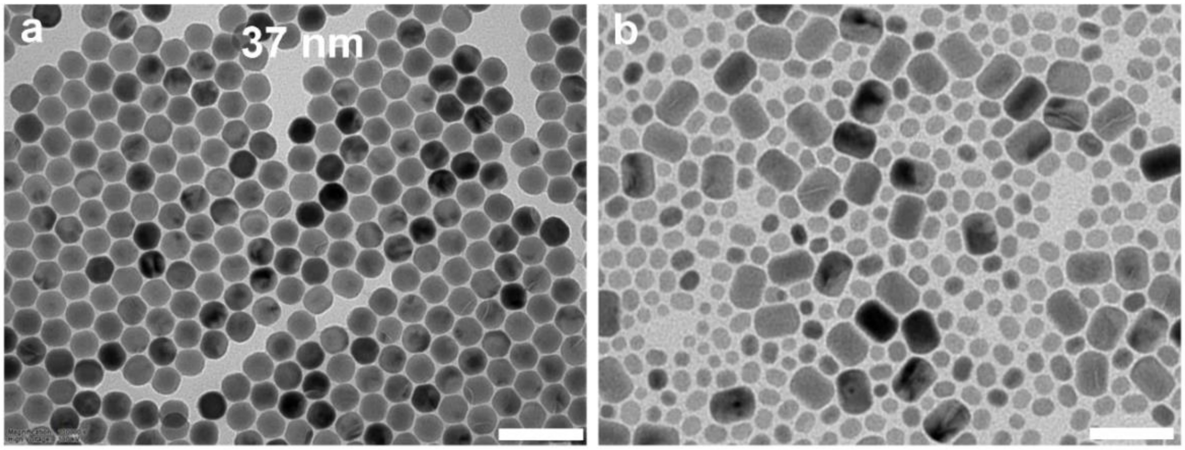

Supplementary Figure 2. TEM images of $NaYF_4$ core nanoparticles (**a**) and $NaYF_4$ nanorods (**b**) synthesized using high-concentration shell precursors (1.465 μmol/mL with an injection rate of 0.25 mL/min) for 16 injection cycles, scale bars are 100 nm.

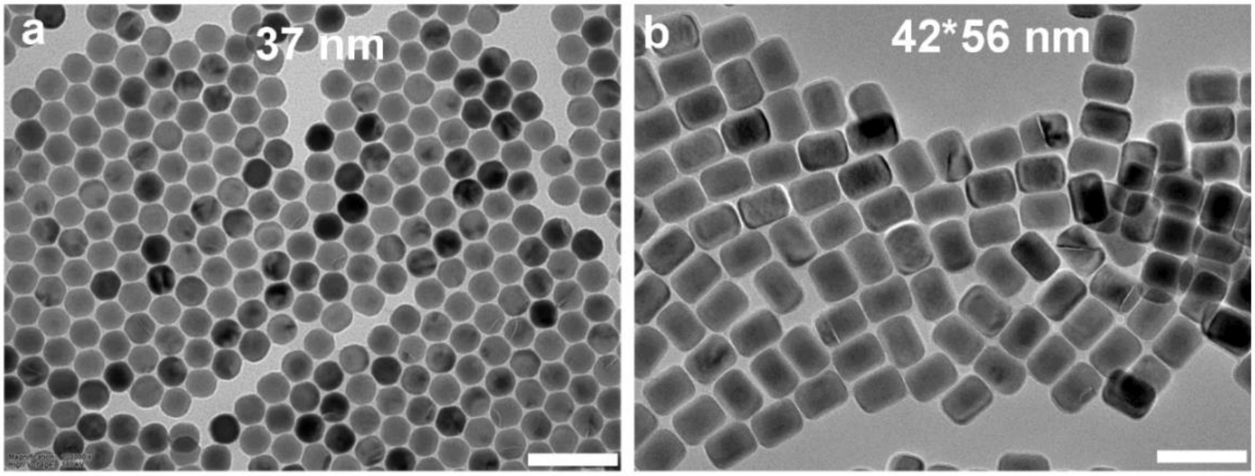

Supplementary Figure 3. TEM images of $NaYF_4$ core nanoparticles (**a**) and $NaYF_4$ nanorods (**b**) synthesized using medium-concentration shell precursors (0.732 μmol/mL with an injection rate of 0.125 mL/min) for 32 injection cycles, scale bars are 100 nm.

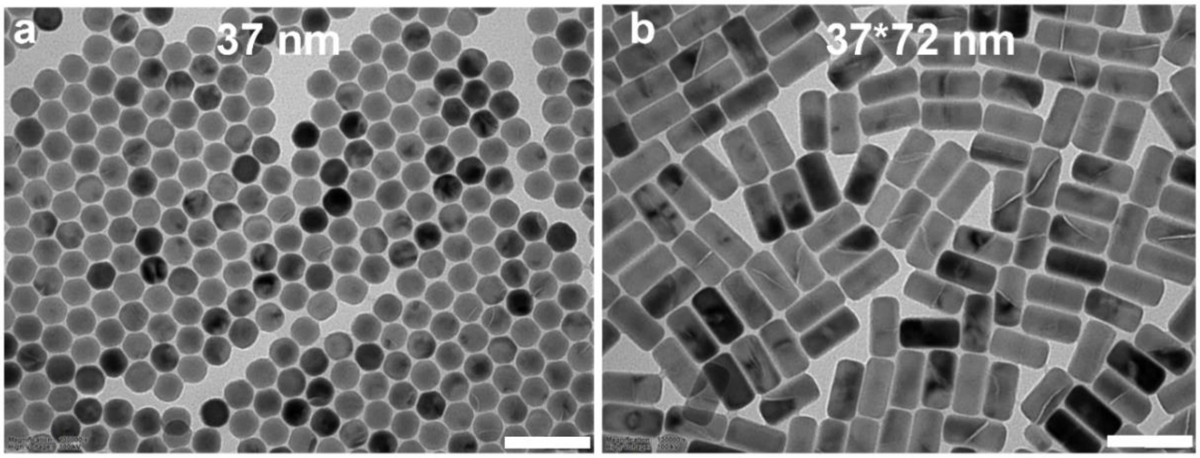

Supplementary Figure 4. TEM images of $NaYF_4$ core nanoparticles (**a**) and $NaYF_4$ nanorods (**b**) synthesized using low-concentration shell precursors (0.235 μmol/mL with an injection rate of 0.04 mL/min) for 100 injection cycles, scale bars are 100 nm.

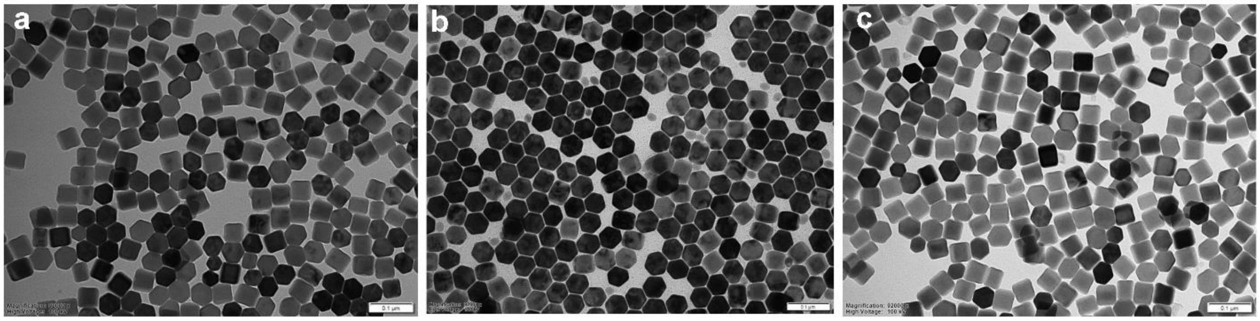

Supplementary Figure 5. TEM images of heterogeneous $NaYF_4$-$NaErF_4$ (a), $NaYF_4$-$NaYbF_4$ (b), and $NaYF_4$-$NaTmF_4$ (c) nanoplates synthesized using a low ratio of OA-/HOA (1:10) and low concentration of shell precursor (0.235 μmol/mL with an injection rate of 0.04 mL/min), scale bars are 100 nm.

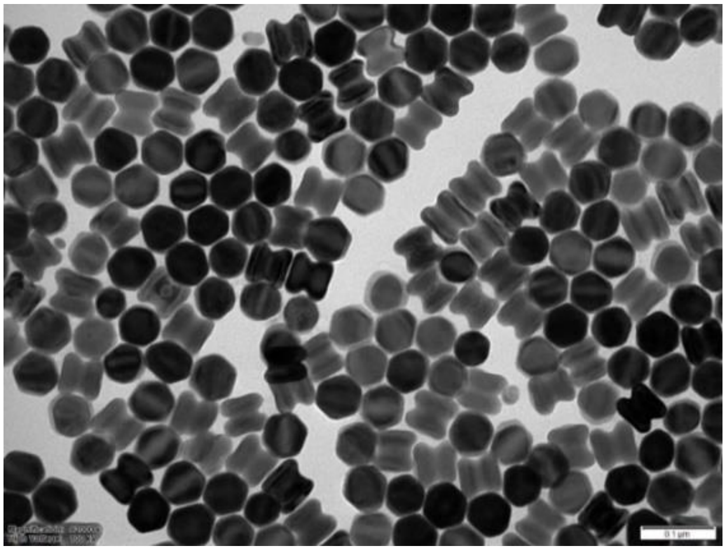

Supplementary Figure 6. TEM image of heterogeneous $NaYF_4$-$NaNdF_4$ nanorods synthesized using a low ratio of OA-/HOA (1:10) and low concentration of shell precursor (0.235 μmol/mL with an injection rate of 0.04 mL/min), scale bar is 100 nm.

Supplementary Note 3. One-dimensional growth of $NaYF_4$ and $NaErF_4$ onto the $NaYF_4$ core nanoparticles.

Heterogeneous upconversion system was frequently employed to tune the emission and excitation wavelengths, the emission intensity and lifetime of the heterogeneous nanocrystals.[2-3] Also, heterogeneous nanostructures are an idea platform to study the crystals growth behaviors. In this study, we choose Y and Er with significant difference in the atomic number to study the heterogeneous growth under one-dimensional control.

Synthesis of 10-segment $NaYF_4$-$NaErF_4$(5nm)-$NaYF_4$(5nm)-$NaErF_4$(5nm)-$NaYF_4$(5nm)-$NaErF_4$ (5nm). The longitudinal growth of $NaYF_4$ and $NaErF_4$ onto the $NaYF_4$ core nanorods was conducted via a successive layer-by-layer hot-injection protocol. Firstly, both $NaYF_4$ and $NaErF_4$ shell precursors were prepared. For $NaErF_4$ shell precursors preparation, 1.5 mmol $ErCl_3$ were added to a 50 mL flask containing 6 mL OA and 15 mL ODE. The mixture was heated to 170 °C under argon for 30 min to obtain a clear solution and then cooled down to about 50 ^{O}C, followed by the addition of 12 mL methanol solution of NH_4F (6.0 mmol), NaOH (3.75 mmol) and KOH (3 mmol). After stirring for 30 min, the solution was heated to 80 °C under argon for 30 min to remove methanol, and then the solution was further heated to 150 °C for another 20 min. Finally, the reaction solution was cooled down to room temperature and labelled as $NaErF_4$ shell precursors.

For the longitudinal growth, 0.2 mmol $NaYF_4$ core particles were added to a 50 mL flask containing 3.6 mL OA, 9.2 mL ODE, and 40 mg NaOH. The mixture was heated to 170 °C under argon for 30 min, and then the solution was further heated to 310 °C . After that, 0.04 mL of $NaYF_4$ shell precursors were injected into the reaction mixture and ripened at 310 °C for 1 min followed by the same injection and ripening cycles for 30 times. Then, 0.04 mL of $NaErF_4$ shell precursors were injected into the reaction mixture and ripened at 310 °C for 1 min followed by the same injection and ripening cycles for another 30 times to get the nanorods with one pairwise $NaErF_4$ segments. For the synthesis of five pairwise $NaYF_4$ and $NaErF_4$ segments ($NaYF_4$-$NaErF_4$-$NaYF_4$-$NaErF_4$-$NaYF_4$-$NaErF_4$) nanorods, the injection process was repeated twice. Finally, the reaction solution was cooled down to room temperature and the formed nanorods were purified according to the procedures used for the purification of $NaYF_4$ core particles. As shown in **Supplementary Figure 7a**, each $NaYF_4$ and $NaErF_4$ segment is clearly observed in the high-angle annular dark-field scanning transmission electron microscopy (HAADF-STEM).

Synthesis of 10-segment $NaYF_4$-$NaErF_4$(10nm)-$NaYF_4$(5nm)-$NaErF_4$(10nm)-$NaYF_4$(5nm)-$NaErF_4$(10nm). 0.2 mmol $NaYF_4$ core particles were added to a 50 mL flask containing 3.6 mL OA, 9.2 mL ODE, and 40 mg NaOH. The mixture was heated to 170 °C under argon for 30 min, and then the solution was further heated to 310 °C. After that, 0.04 mL of $NaYF_4$ shell precursors were injected into the reaction mixture and ripened at 310 °C for 1 min followed by the same injection and ripening cycles for 30 times. Then, 0.04 mL of $NaErF_4$ shell precursors were injected into the reaction mixture and ripened at 310 °C for 1 min followed by the same injection and ripening cycles for another 60 times to get the nanorods with one pairwise $NaErF_4$ segments. For the synthesis of five pairwise $NaYF_4$ and $NaErF_4$ segments ($NaYF_4$-$NaErF_4$-$NaYF_4$-$NaErF_4$-$NaYF_4$-$NaErF_4$) nanorods, the injection process was repeated twice. Finally, the reaction solution was cooled down to room temperature and the formed nanorods were purified according to the procedures used for the purification of $NaYF_4$ core particles.

Synthesis of 18-segment $NaYF_4$-$NaErF_4$(5nm)-$NaYF_4$(5nm)-$NaErF_4$(5nm)-$NaYF_4$(5nm)-$NaErF_4$(5nm). 0.2 mmol $NaYF_4$ core particles were added to a 50 mL flask containing 3.6 mL OA, 9.2 mL ODE, and 40 mg NaOH. The mixture was heated to 170 °C under argon for 30 min, and then the solution was further heated to 310 °C. After that, 0.04 mL of $NaYF_4$ shell precursors were injected into the reaction mixture and ripened at 310 °C for 1 min followed by the same injection and ripening cycles for 30 times. Then, 0.04 mL of $NaErF_4$ shell precursors were injected into the reaction mixture and ripened at 310 °C for 1 min followed by the same injection and ripening cycles for another 30 times to get the nanorods with one pairwise $NaErF_4$ segments. For the synthesis of nine pairwise $NaYF_4$ and $NaErF_4$ segments ($NaYF_4$-$NaErF_4$-$NaYF_4$-$NaErF_4$-$NaYF_4$-$NaErF_4$-$NaYF_4$-$NaErF_4$-$NaYF_4$-$NaErF_4$) nanorods, the injection process was repeated four times. Finally, the reaction solution was cooled down to room temperature and the formed nanorods were purified according to the procedures used for the purification of $NaYF_4$ core particles. As shown in **Supplementary Figure 7b**, each $NaYF_4$ and $NaErF_4$ segment is clearly observed in the high-angle annular dark-field scanning transmission electron microscopy. Also, it is notable that the widths of the nanorods are the same even if the length and the number of the segments are different (**Supplementary Figure 7c,d**), which is indicative of the one-dimensional growth of the heterogeneous nanocrystals.

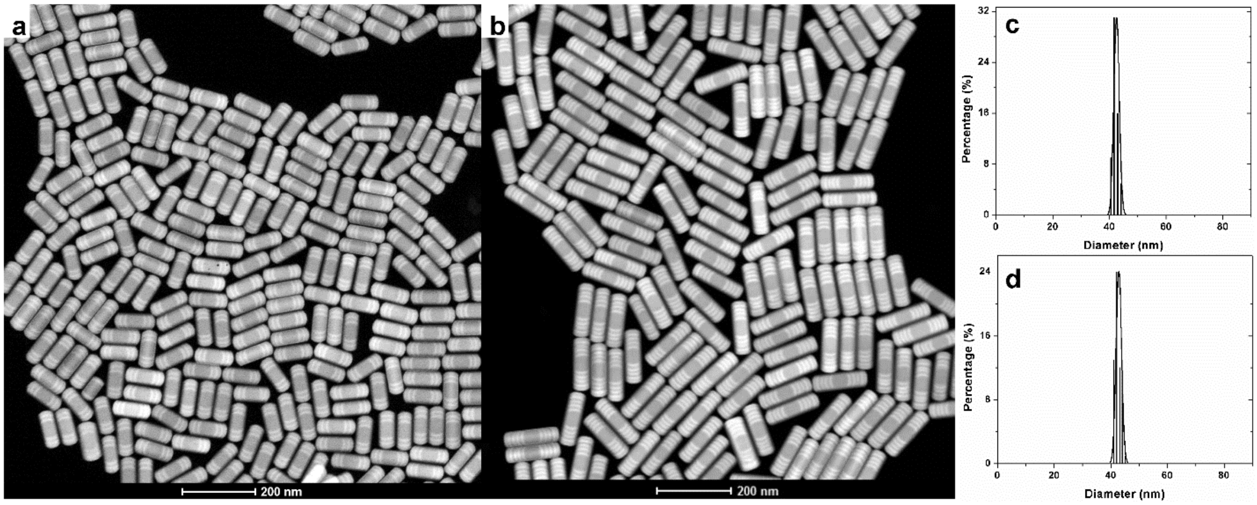

Supplementary Figure 7. High-angle annular dark-field scanning transmission electron microscopy (HAADF-STEM) images (**a**,**b**) and width distribution histograms (**c**,**d**) of typical heterogeneous $NaYF_4$-$NaErF_4$-$NaYF_4$-$NaErF_4$-$NaYF_4$-$NaErF_4$ (**a**,**c**) and $NaYF_4$-$NaErF_4$-$NaYF_4$-$NaErF_4$-$NaYF_4$-$NaErF_4$-$NaYF_4$-$NaErF_4$-$NaYF_4$-$NaErF_4$ (**b**,**d**) nanorods.

Supplementary Note 4. Design and synthesis of optical nanorods with different length

Following the advantages of the heterogeneous nanocrystal control and the super-resolution imaging, we further fabricate different batches of highly-uniform and distance-tunable optical nanorods (**Supplementary Figure 9**). By converting the negative contrast images into the positive super-resolution images (**Supplementary Figure 10 and 11**), the uniformity of five representative kinds of nanorods with the distances of active sections from 76 to 141 nm are summarized, which optical results are highly consistent to the TEM characterizations.

Synthesis of inert $NaYF_4$ nanorods. Controlled one-dimensional growth of $NaYF_4$ core nanoparticles was conducted *via* a successive layer-by-layer hot-injection protocol. 0.2 mmol $NaYF_4$ core particles were added to a 50 mL flask containing 3.6 mL OA, 9.2 mL ODE, and 40 mg NaOH. The mixture was heated to 170 °C under argon for 30 min, and then the solution was further heated to 310 °C. After that, 0.04 mL of $NaYF_4$ shell precursors were injected into the reaction mixture and ripened at 310 °C for 1 min followed by the same injection and ripening cycles for different times to get the nanorods with different lengths. Finally, the formed nanorods were purified for TEM characterization. **Supplementary Figure 9** shows the TEM results of a series of uniform $NaYF_4$ nanorods with the tunable length (**X**) from 55 nm to 120 nm.

Synthesis of $NaYF_4$-$NaYF_4$:Yb,Tm nanorods with different length. The $NaYF_4$ nanorods with different lengths were used as the core and added to a 50 mL flask containing 3.6 mL OA, 9.2 mL ODE, and 40 mg NaOH. The mixture was heated to 170 °C under argon for 30 min, and then the solution was further heated to 310 °C. After that, 0.04 mL of $NaYF_4$:40%Yb,4%Tm shell precursors were injected into the reaction mixture and ripened at 310 °C for 1 min followed by the same injection and ripening cycles for another 110 times to get the nanorods with one pairwise $NaYF_4$:40%Yb,4%Tm segments. Finally, the reaction solution was cooled down to room temperature, and the formed nanorods were precipitated by ethanol and washed with cyclohexane, ethanol and methanol for 3 times to get the $NaYF_4$-$NaYF_4$:40%Yb,4%Tm nanorods with different lengths (**Y**) from 95 nm to 160 nm. Therefore, the center-to-center distances of the rods (**Z**) are equal to (**X**+**Y**)/2 as shown in **Supplementary Figure 9** and these distances are highly consistent to the results of the super-resolution characterizations.

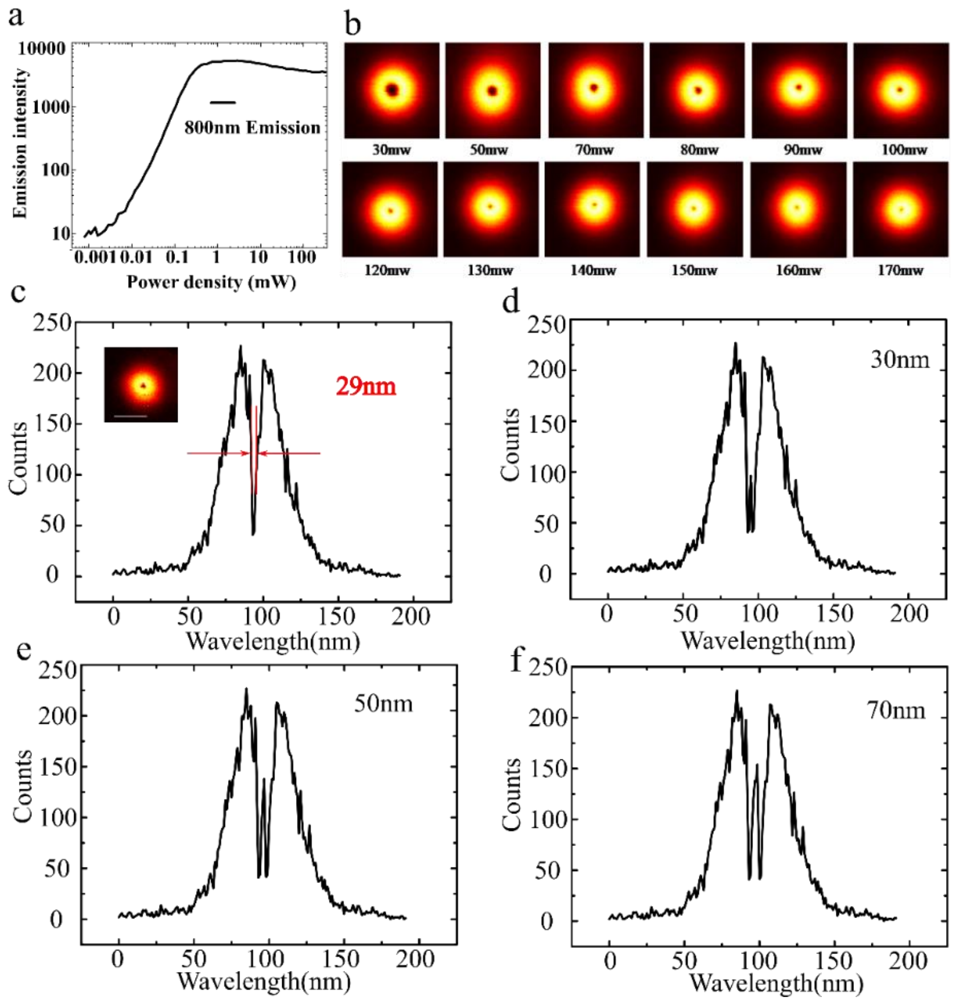

Supplementary Figure 8. (**a**) The saturation intensity curve of the 800 nm emissions from single nanoparticles ($NaYF_4$:40%Yb^{3+},4%Tm^{3+}) under 980 nm excitation. (**b**) Negative images of single particle under different excitation powers. (**c**) The corresponding cross-section profile in figure b, insert shows the negative-shaped PSF of single nanoparticle under the excitation power of 80 mW. (**d-f**) Merged PSF with different central shift distance of 30 nm, 50 nm, and 70 nm respectively. Pixel dwells time is 3 ms; all the pixel size is 10 nm. Scale bar is 500 nm.

The FWHM of the PSF with a dip of our optical system is 29 nm, which can be defined as the experiment resolution. In this case, two active parts with distance from 30 to 70 nm has been simulated, where we overlap two experimental PSF. When the distance is 30 nm, the two active segments cannot be well resolved as the intensity of the central spot (spot 2 in Fig. 2c) is only slightly higher than that of the two negative parts (spots 1 and 3 in Fig. 2c). For two parts with a spacing of 50 nm, they can be theoretically resolved.

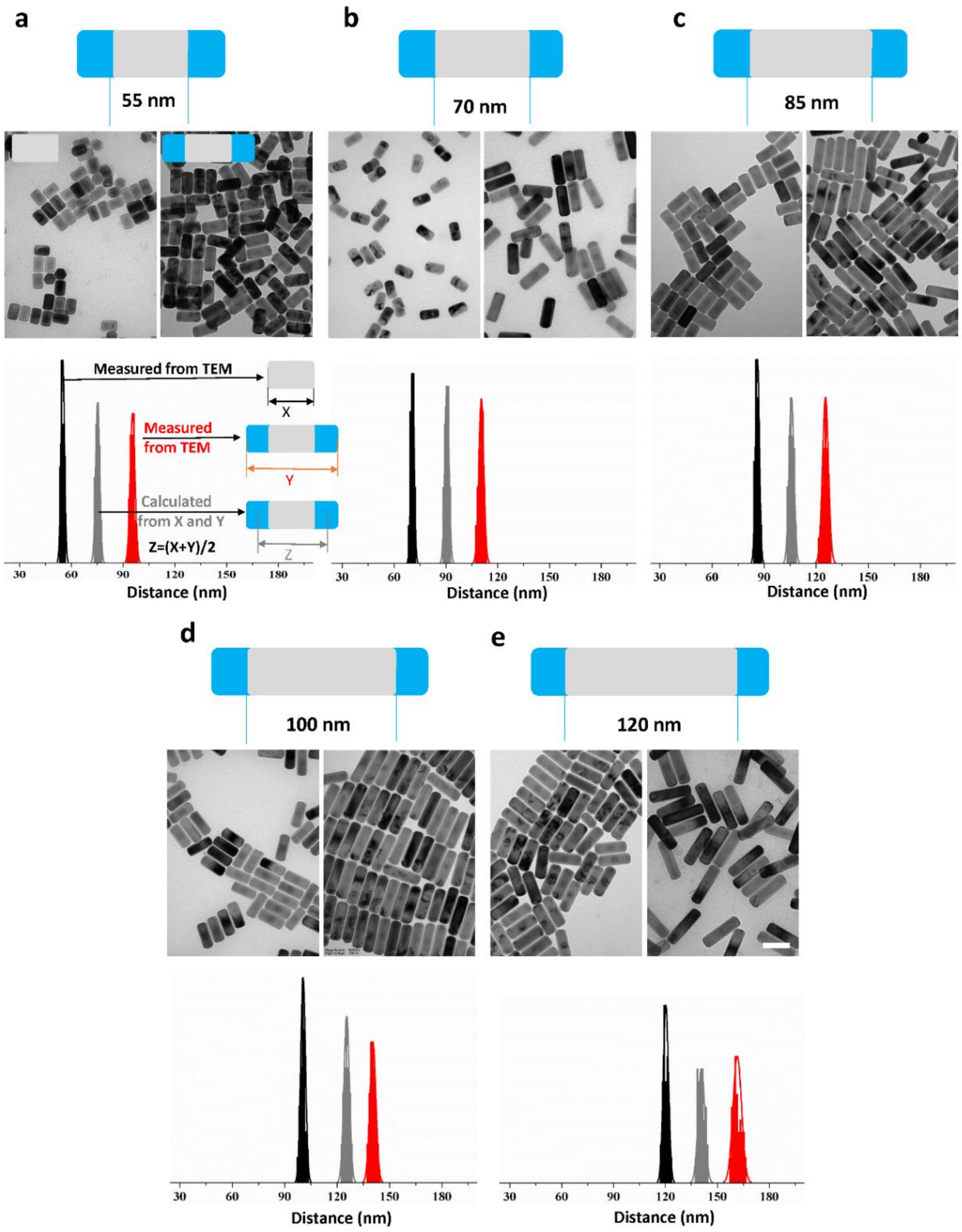

Supplementary Figure 9. Design structure, TEM images, distance distributions of a series of heterogeneous upconversion nanorods with tunable lengths of the inert section to separate the optically active components on the ends, **X** represents the distance of the two ends of the inert section, **Y** represents the distance of the two ends of the actively doped part, **Z** represents the center-to-center distance of the two actively doped parts, scale bar is 100 nm.

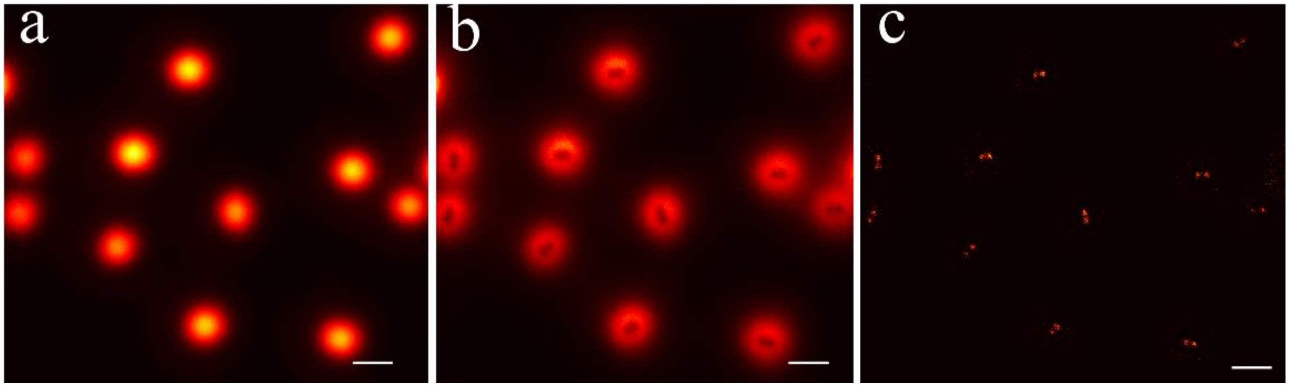

Supplementary Figure 10. Illustration of the positive-contrast super-resolution imaging process. (**a**) Confocal image of upconversion nanorods. (**b**) Negative-contrast super-resolution image of upconversion nanorods. (**c**) The processed positive-contrast super-resolution image of upconversion nanorods. Dwell time is 2 ms. Scale bar is 500 nm.

Here we use a subtraction algorithm to obtain the deconvoluted super-resolution images.[7] Supplementary Figure 10 shows the key process based on two images, i.e. the "negative-contrast image" captured by scanning the sample by doughnut beam (Supplementary Figure 10b), and the "confocal image" generated by either scanning the sample by Gaussian beam or applying a low pass image filter on the "negative-contrast image" (Supplementary Figure 10a). The final positive-contrast super-resolution image (Supplementary Figure 10c) is constructed by the intensity subtraction of the two images. We use equation 1 to illustrate the subtraction process.

$$I_{pos}=I_{con}-r\times I_{neg} \qquad (1)$$

Where I_{con}, I_{neg}, and I_{pos} are the normalized intensity distributions of the confocal, negative-contrast super-resolution image, and positive-contrast image, respectively. r is the subtractive factor used to avoid the negative values to improve the image quality.

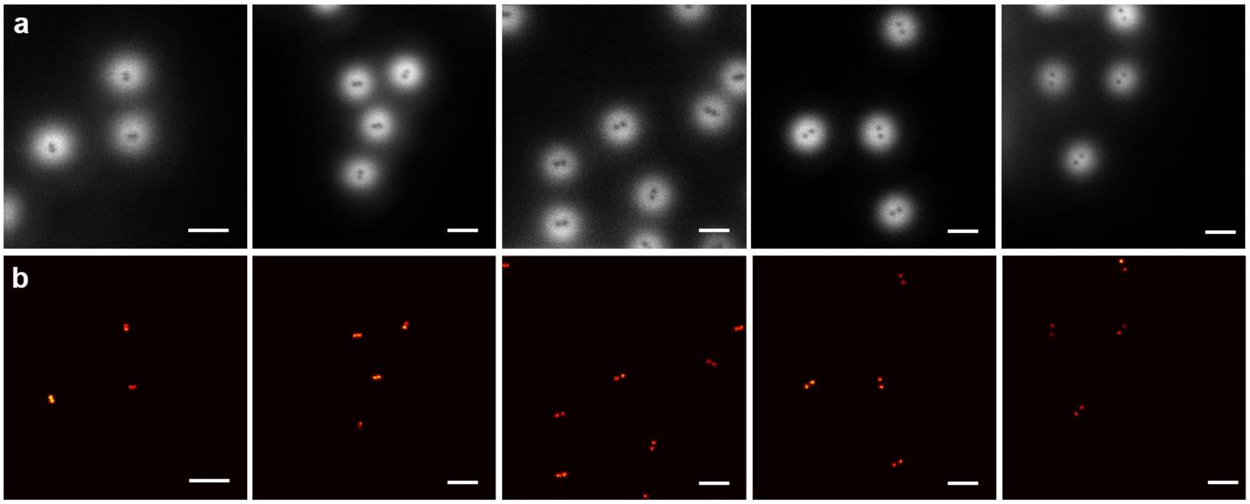

Supplementary Figure 11. Large-area negative-contrast images (**a**), and positive super-resolution images (**b**) of a series of heterogeneous upconversion nanorods corresponding with that in Supplementary Figure 9 and fig. 2d. Scale bars are 500 nm.

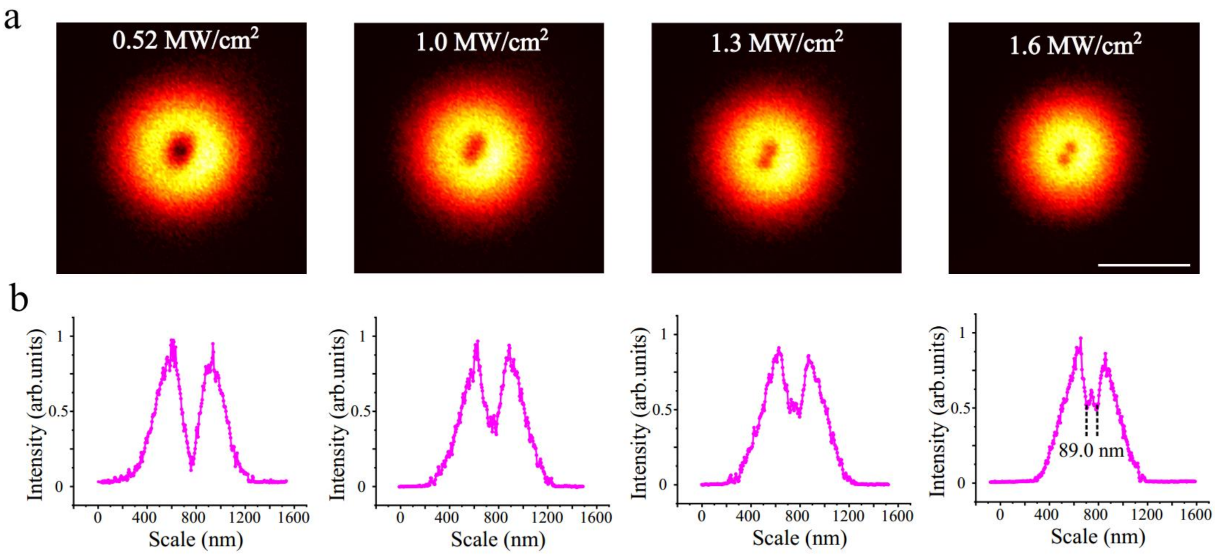

Supplementary Figure 12. (**a**) “Negative” images of the cross-section profiles of a single nanorod under different excitation powers. (**b**) Cross-section profiles of upconversion emission of nanorods in **a** at four different excitation powers. Pixel size, 10 nm. Scale bar is 500 nm, dwell time is 1 ms.

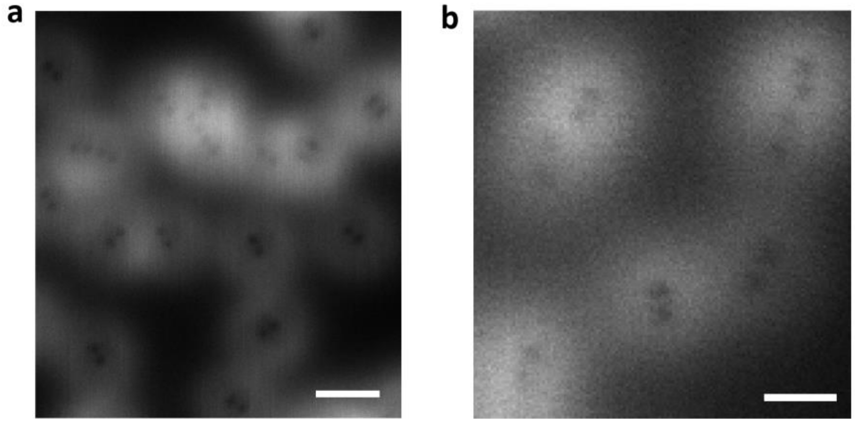

Supplementary Figure 13. Negative super-resolution images of the high concentration nanorods with the same sample (**a**) and the mixture of two kinds of nanorods (**b**). Scale bars in **a** and **b** are 400 and 250 nm, respectively.

Supplementary Note 5. Design and synthesis of colour-based nanorods

With well controlled heterogeneous nanostructures, more complexed optical nanopatterns can be achieved by measuring the unique optical properties of upconversion nanorods. Emission wavelength coding (colour coding) is commonly used for the construction of most existing fluorescent tags for optical multiplexing. We first show the formation of heterogeneous nanorods with different emissions through doping of Tm and Er at different positions of the rods, as shown in **Supplementary Figure 14**.

Synthesis of color-based nanorods. For color-based nanorod fabrication, $NaYF_4$ nanorods with the length of 85 nm were first synthesized. Then the $NaYF_4$ nanorods were used as the core and added to a 50 mL flask containing 3.6 mL OA, 9.2 mL ODE, and 40 mg NaOH. The mixture was heated to 170 °C under argon for 30 min, and then the solution was further heated to 310 °C. After that, 0.04 mL of $NaYF_4$:40%Yb,4%Tm shell precursors were injected into the reaction mixture and ripened at 310 °C for 1 min followed by the same injection and ripening cycles for 110 times to get the nanorods with the NIR-light emitting sections. Then, 0.04 mL of $NaYF_4$ shell precursors were injected into the reaction mixture and ripened at 310 °C for 1 min followed by the same injection and ripening cycles for 15 times to get the nanorods with the inert sections. Finally, 0.04 mL of $NaYF_4$:40%Yb,6%Er shell precursors were injected into the reaction mixture and ripened at 310 °C for 1 min followed by the same injection and ripening cycles for 110 times to get the nanorods with the red-light emitting sections.

Characterization of color-based nanorods. The formed nanorods were purified for TEM characterization. As shown in **Supplementary Figure 14a**, we can clearly see that the Yb/Tm and Yb/Er co-doped sections through the HAADF-STEM image, which is consistent with our design. As shown in the super-resolution imaging (**Supplementary Figure 14b-c**), the optical emissions in different colour channels for different sections could be well resolved with high uniformity.

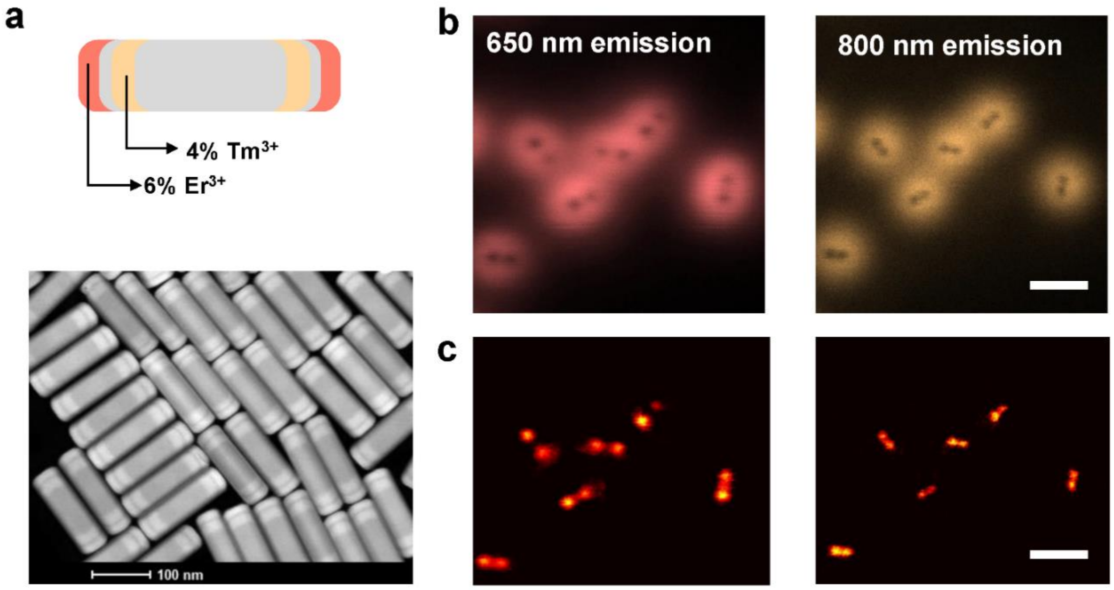

Supplementary Figure 14. (a) Structure design and HAADF-STEM images of heterogeneous nanorods with emitters Tm and Er doped at different positions for colour barcoding. Negative (**b**) and positive (**c**) super-resolution images of the fabricated nanorods in different channels of emission wavelength. Scale bars in **b** and **c** are 500 nm.

Supplementary Note 6. Design and synthesis of excitation-power-based nanorods

Not only two sections with different doped emitters and emission colours could be decoded, but also the same emitters with same colour but only slightly different doping concentrations could be discerned due to the power-dependent properties of upconversion nanorods.[4-5] For the first time, we show the formation of heterogeneous nanorod with different power-dependent properties through the doping of different concentrations of Tm at different position of the rods, as shown in **Supplementary Figure 15**.

Synthesis of excitation-power-based nanorods. For excitation-power-based nanorods fabrication, $NaYF_4$ nanorods with the length of 85 nm were first synthesized. Then the $NaYF_4$ nanorods were used as the core and added to a 50 mL flask containing 3.6 mL OA, 9.2 mL ODE, and 40 mg NaOH. The mixture was heated to 170 °C under argon for 30 min, and then the solution was further heated to 310 °C. After that, 0.04 mL of $NaYF_4$:40%Yb,4%Tm shell precursors were injected into the reaction mixture and ripened at 310 °C for 1 min followed by the same injection and ripening cycles for 110 times. Then, 0.04 mL of $NaYF_4$ shell precursors were injected into the reaction mixture and ripened at 310 °C for 1 min followed by the same injection and ripening cycles for 15 times to get the nanorods with the inner sections. Finally, 0.04 mL of $NaYF_4$:40%Yb,10%Tm shell precursors were injected into the reaction mixture and ripened at 310 °C for 1 min followed by the same injection and ripening cycles for 110 times to form the excitation-power-based nanorods.

Characterization of excitation-power-based nanorods. The formed nanorods were purified for TEM characterization. As shown in **Supplementary Figure 15a**, the formed nanorods are uniform in size and morphology. Also, the super-resolution images (**Supplementary Figure 15b**) indicated the optical emission pattern under different excitation power could be well resolved with high uniformity. Two channels in the excitation power dimension were well consistent with our design and demonstrated the different distances of the doped parts. This result illustrates the potential of this untapped dimension independent of the conventional emission channels for optical multiplexing.

Characterization of lifetime-based nanorods. As the lifetime of the upconversion luminescence is highly depend on the doping concentration of the emitters, the difference in doping concentration could be also used in lifetime-based multiplexing. As shown in **Supplementary Figure 16**, we can get the optical emissions with same kinds of dopants (Yb/Tm) at different sections by time-gated super-resolution imaging.

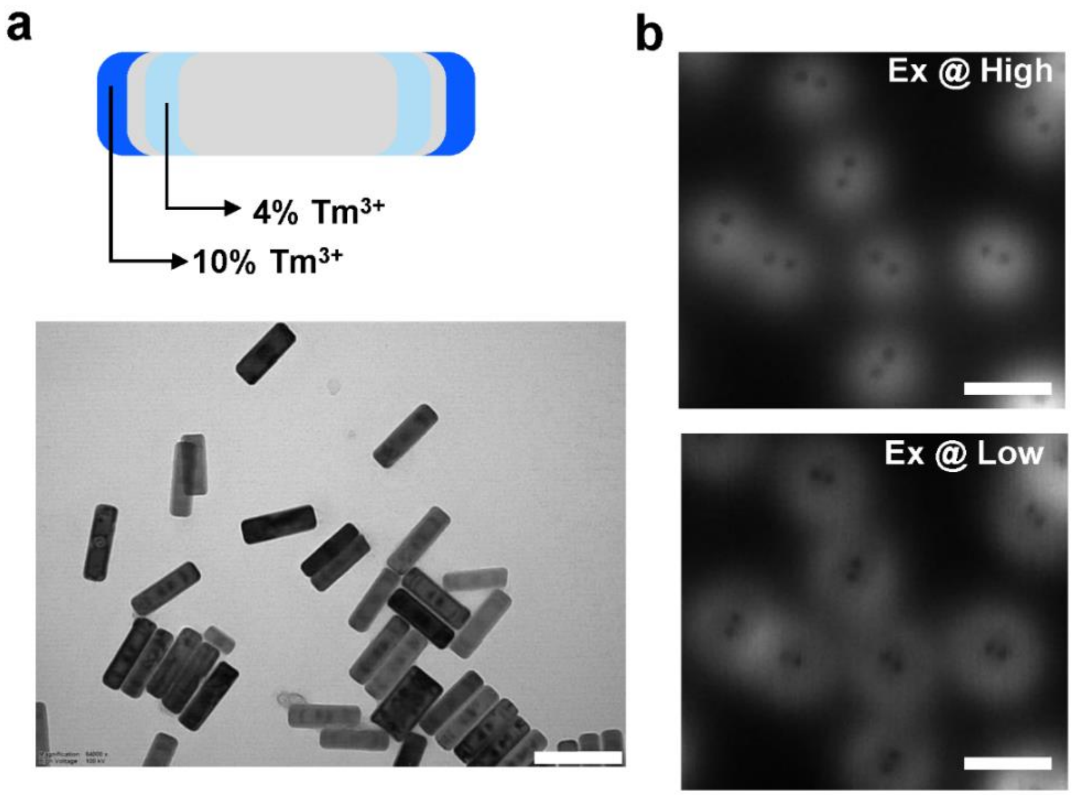

Supplementary Figure 15. (a) Structure design and TEM images of the heterogeneous nanorods with different Tm doping concentrations at different positions for power-dependent barcoding, scale bar is 200 nm. **(b)** Negative super-resolution images of the formed nanorods under different excitation powers, scale bars are 500 nm.

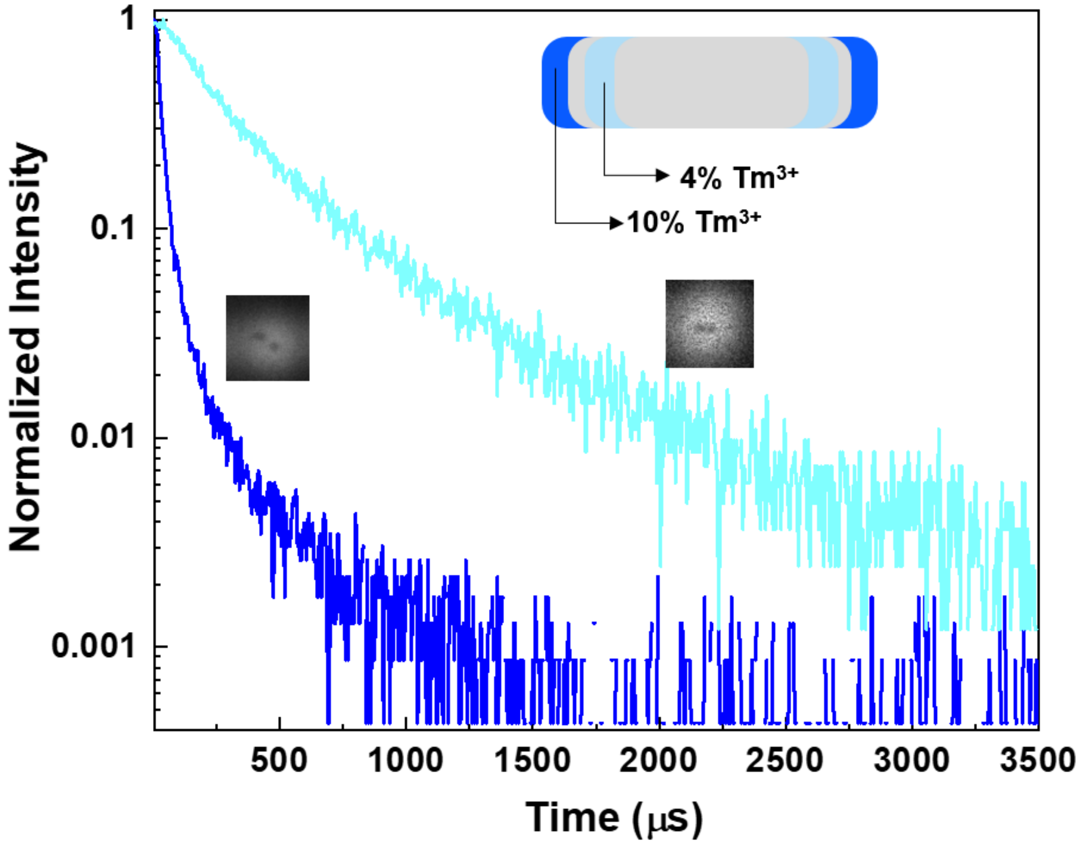

Supplementary Figure 16. Illustration of time-gated super-resolution images of heterogeneous nanorods with different concentrations (4% and 10%) of Tm under pulsed illumination. Luminescence decay curves at 800 nm shown that 4% Tm doped sample has the much longer lifetime than that from 10% Tm doped sample.

Supplementary Note 7. Design and synthesis of excitation-wavelength-based nanorods

Controlled energy migration in the core@shell UCNPs systems enables the fine-tuning of both upconversion emission colours and excitation wavelength.[2, 6] Encouragingly, we show here that the excitation wavelength could be used as fourth dimension for the rod coding. In a very simple design, we have one emission part (Yb/Er), packed closely with Yb/Nd section (**Supplementary Figure 17**). Therefore, 980 nm laser could directly excite the emission parts with Yb doping, while 808 nm laser can excite the Nd in Nd/Yb sections followed by the energy transfer and migration to the emission parts ($Nd^{3+} \rightarrow Yb^{3+} \rightarrow Yb^{3+} \rightarrow Er^{3+}$). It should be noted that the super-resolution signals will appear at the absorption parts instead of the emission parts in the energy migration upconversion system. Therefore, we can easily decoding the close packed absorption parts with different excitation wavelengths.

Synthesis of excitation-wavelength-based nanorods. For excitation-wavelength-based nanorod fabrication, $NaYF_4$ nanorods with the length of 120 nm were first synthesized. Then the $NaYF_4$ nanorods were used as the core and added to a 50 mL flask containing 3.6 mL OA, 9.2 mL ODE, and 40 mg NaOH. The mixture was heated to 170 °C under argon for 30 min, and then the solution was further heated to 310 °C. After that, 0.04 mL of $NaYF_4$:20%Yb,60%Nd shell precursors were injected into the reaction mixture and ripened at 310 °C for 1 min followed by the same injection and ripening cycles for 30 times. Then, 0.04 mL of $NaYF_4$:40%Yb,6%Er shell precursors were injected into the reaction mixture and ripened at 310 °C for 1 min followed by the same injection and ripening cycles for 80 times to get the excitation-wavelength-based nanorods.

Characterization of excitation-wavelength-based nanorods. The formed nanorods were purified for TEM characterization. As shown in **Supplementary Figure 17a**, the formed nanorods are uniform in size and the morphology. Also, the super-resolution images (**Supplementary Figure 17b**) indicated the optical emission patterns under different excitation wavelengths could be well resolved with high uniformity. Two channels in the excitation wavelength dimension were well consistent with our design and demonstrated the different distances of the sensitizer-doped parts. This result illustrates the potential of this untapped dimension independent of the conventional emission channels for optical multiplexing.

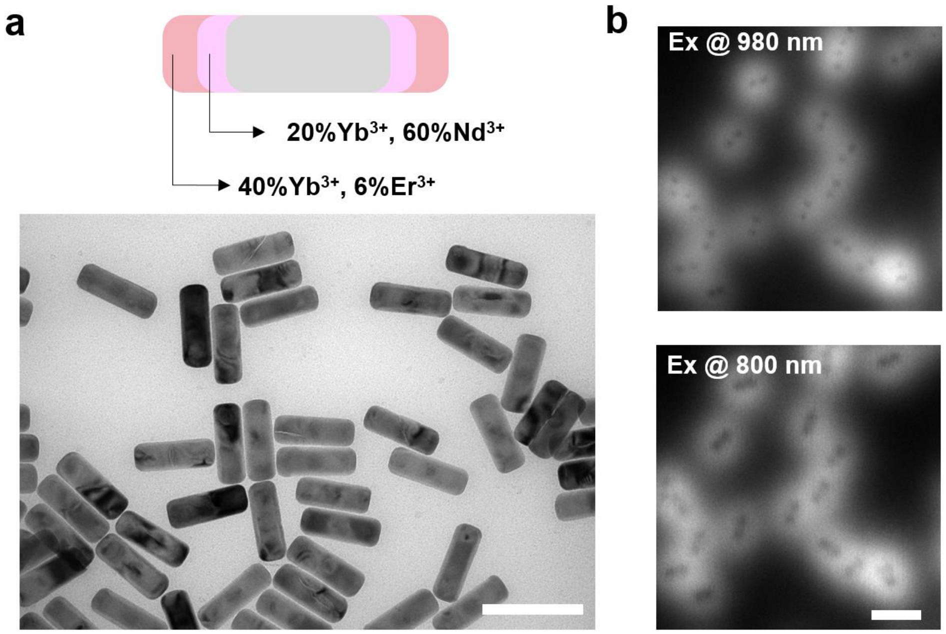

Supplementary Figure 17. (a) Structure design and TEM image of the heterogeneous nanorods with Nd/Yb-Yb/Er doping sections at different positions for excitation-wavelength-dependent barcoding, scale bar is 200 nm. (**b)** Negative super-resolution images of the formed nanorods under different excitation wavelengths, scale bar is 500 nm.

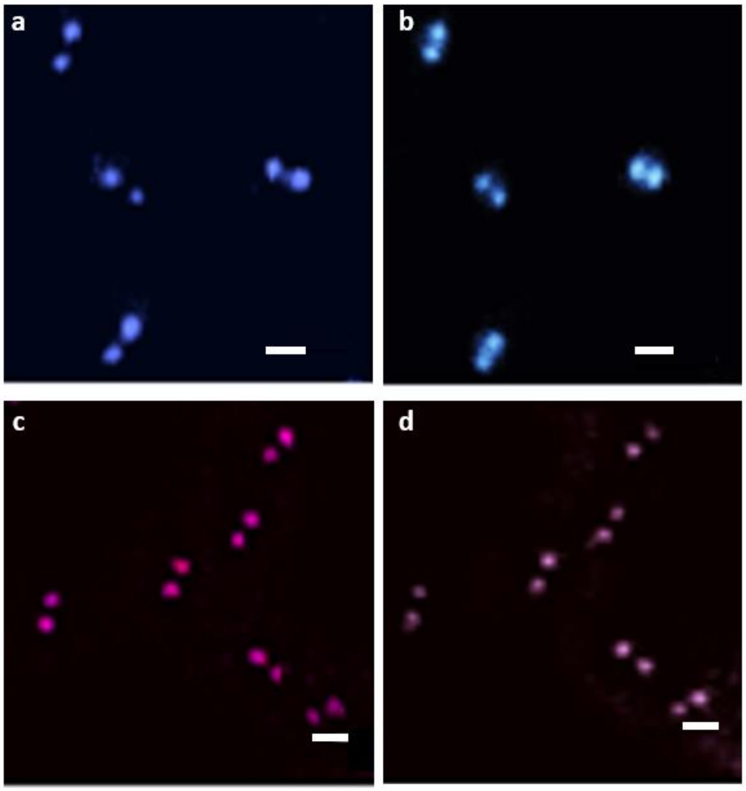

Supplementary Figure 18. High-resolution images of the functional segments selectively decoded by high power density (**a**), low power density (**b**), 980 nm excitation (**c**) and 808 nm excitation (**d**). Scale bar are 200 nm. Large area images are shown in figure 3 **b** and **f.**

Supplementary Note 8. Design and synthesis of RGB colors nanorods.

The ability of selective activations of high-dimensional emission diversities from a single nanorod can further create a sub-diffraction-limit RGB-switchable pixel, on demand and in response to the specific excitation wavelength and donut illumination patterns.

Synthesis of RGB colors nanorods. For RGB colors nanorod fabrication, $NaYF_4$:15%Er nanoparticles were first synthesized. Then 0.2 mmol $NaYF_4$:15%Er nanoparticles were used as the core and added to a 50 mL flask containing 3.6 mL OA, 9.2 mL ODE, and 40 mg NaOH. The mixture was heated to 170 °C under argon for 30 min, and then the solution was further heated to 310 °C. After that, 0.04 mL of $NaYF_4$ shell precursors were injected into the reaction mixture and ripened at 310 °C for 1 min followed by the same injection and ripening cycles for 240 times. Then, 0.04 mL of $NaYF_4$:40%Yb,4%Tm shell precursors were injected into the reaction mixture and ripened at 310 °C for 1 min followed by the same injection and ripening cycles for 110 times. Followed, 0.04 mL of $NaYF_4$ shell precursors were injected into the reaction mixture and ripened at 310 °C for 1 min followed by the same injection and ripening cycles for another 240 times. Finally, 0.04 mL of $NaYF_4$:30%Yb,2%Er shell precursors were injected into the reaction mixture and ripened at 310 °C for 1 min followed by the same injection and ripening cycles for 110 times to get the RGB rods.

Supplementary References

1. Liu, D.; Xu, X.; Du, Y.; Qin, X.; Zhang, Y.; Ma, C.; Wen, S.; Ren, W.; Goldys, E. M.; Piper, J. A.; Dou, S.; Liu, X.; Jin, D., Three-dimensional controlled growth of monodisperse sub-50 nm heterogeneous nanocrystals. *Nat. Commun.* **2016,** *7*, 10254.
2. Wang, F.; Liu, X., Multicolor Tuning of Lanthanide-Doped Nanoparticles by Single Wavelength Excitation. *Acc. Chem. Res.* **2014,** *47* (4), 1378-1385.
3. Fan, Y.; Wang, P.; Lu, Y.; Wang, R.; Zhou, L.; Zheng, X.; Li, X.; Piper, J. A.; Zhang, F., Lifetime-engineered NIR-II nanoparticles unlock multiplexed in vivo imaging. *Nat. Nanotech.* **2018,** *13* (10), 941-946.
4. Wang, F.; Wen, S.; He, H.; Wang, B.; Zhou, Z.; Shimoni, O.; Jin, D., Microscopic inspection and tracking of single upconversion nanoparticles in living cells. *Light Sci. Appl.* **2018,** *7*, e18007.
5. Gargas, D. J.; Chan, E. M.; Ostrowski, A. D.; Aloni, S.; Altoe, M. V. P.; Barnard, E. S.; Sanii, B.; Urban, J. J.; Milliron, D. J.; Cohen, B. E.; Schuck, P. J., Engineering bright sub-10-nm upconverting nanocrystals for single-molecule imaging. *Nat. Nanotech.* **2014,** *9* (4), 300-305.
6. Wang, Y.-F.; Liu, G.-Y.; Sun, L.-D.; Xiao, J.-W.; Zhou, J.-C.; Yan, C.-H., Nd3+-Sensitized Upconversion Nanophosphors: Efficient In Vivo Bioimaging Probes with Minimized Heating Effect. *ACS Nano* **2013,** *7* (8), 7200-7206.
7. Kuang, C.; Li, S.; Liu, W.; Hao, X.; Gu, Z.; Wang, Y.; Ge, J.; Li, H.; Liu, X., Breaking the Diffraction Barrier Using Fluorescence Emission Difference Microscopy. *Scientific Reports* **2013,** *3* (1), 1441.
